# Stability of variable importance scores and rankings using statistical learning tools on single-nucleotide polymorphisms and risk factors involved in gene × gene and gene × environment interactions

**DOI:** 10.1186/1753-6561-1-s1-s58

**Published:** 2007-12-18

**Authors:** Kristin K Nicodemus, Wenyi Wang, Yin Yao Shugart

**Affiliations:** 1 Wellcome Trust Centre for Human Genetics, Roosevelt Drive, Oxford, OX3 7BN, UK; 2 Department of Clinical Pharmacology, University of Oxford, Woodstock Road, Oxford, OX2 6HA, UK; 3 Stanford Genome Technology Center, 855 California Avenue, Palo Alto, CA 94304, USA; 4 Department of Epidemiology, Johns Hopkins Bloomberg School of Public Health, 615 North Wolfe Street, Baltimore, Maryland 21205, USA

## Abstract

Risk of complex disorders is thought to be multifactorial, involving interactions between risk factors. However, many genetic studies assess association between disease status and markers one single-nucleotide polymorphism (SNP) at a time, due to the high-dimensional nature of the search space of all possible interactions. Three ensemble methods have been recently proposed for use in high-dimensional data (Monte Carlo logic regression, random forests, and generalized boosted regression). An intuitive way to detect an association between genetic markers and disease status is to use variable importance measures, even though the stability of these measures in the context of a whole-genome association study is unknown. For the simulated data of Problem 3 in the Genetic Analysis Workshop 15 (GAW15), we examined the variability of both rankings and magnitude of variable importance measures using 10 variables simulated to participate in gene × gene and gene × environment interactions. We conducted 500 analyses per method on one randomly selected replicate, tallying the rankings and importance measures for each of the 10 variables of interest. When the simulated effect size was strong, all three methods showed stable rankings and estimates of variable importance. However, under conditions more commonly expected to be encountered in complex diseases, random forests and generalized boosted regression showed more stable estimates of variable importance and variable rankings. Individuals endeavoring to apply statistical learning methods to detect interaction in complex disease studies should perform repeated analyses in order to assure variable importance measures and rankings do not vary greatly, even for statistical learning algorithms that are thought to be stable.

## Background

The use of statistical learning methods to detect interactions between genetic and environmental risk factors is fuelled by the necessity of using methods developed for use with high-dimensional data (e.g., whole-genome association studies (WGAs)), in which explicitly considering all possible two-way, three-way, or higher-order interactions is computationally not feasible. Ensemble methods, or methods that consider multiple models such as classification trees, may be a more efficient way to detect interactions in a high-dimensional space. A simple and intuitive approach for selection of 'interesting' variables that may be involved in interaction is to use the associated importance measure for each variable to rank them (e.g., [1]) and to prioritize variables ranked as having high importance scores for further study. However, in the context of a WGA study, the variability in rankings and importance scores of machine learning methods is unknown. Using the Genetic Analysis Workshop 15 (GAW15) data simulated to mimic a genome-wide association study of rheumatoid arthritis (RA), we tested three statistical learning tools to assess variability in rankings and importance scores within each method on variables simulated as participating in gene × gene or gene × environment interaction.

The statistical methods assessed in this study included Monte Carlo logic regression (MCLR; using the R [2]**LogicReg **package) [3-6], random forests (RF; using the R package **randomForest**) [7-9], and generalized boosted regression (using the R **gbm **package) [10-12]. Logic regression constructs Boolean combinations of binary variables (such as single-nucleotide polymorphisms (SNPs) in dominant and recessive coding) in a regression framework [1,2]. In contrast to selecting a 'best fitting' logic tree, Monte Carlo logic regression extends this approach, and tallies single variables and higher order variable interactions, investigated during the run of a homogeneous Markov chain, which can be considered a measure of variable importance [3]. The random forest approach is a classification-tree method that creates an ensemble of trees, generated by bootstrap samples of the data and randomly selected subsets of the predictors. This "random forest" of trees uses a consensus vote to predict the outcome. A measure of variable importance (using the Gini impurity index) is calculated using the independent or "out-of-bag" samples. Finally, we used the generalized boosted regression method (implemented in the R package **gbm**) with decision stumps (decision trees with a single split) as the base classifier. Boosting algorithms can be seen as gradient-descent algorithms that seek to find a weak classifier that most reduces the error or loss function [11]. The contribution of the predictors for reduction of the deviance was used as a measure of variable importance.

## Methods

Because the focus of this study was to examine the stability of variable importance rankings and scores for three ensemble methods on variables involved in simulated interactions, we requested the generating model for the simulated data before beginning analysis. After reviewing the generating model, we included data from all chromosomes containing SNPs simulated with gene × gene or gene × environment interaction: chromosome 6 (674 SNPs), chromosome 8 (442 SNPs), chromosome 16 (204 SNPs), plus lifetime smoking status and sex. On chromosomes 16 and 8 no linkage disequilibrium (LD) was simulated between the disease loci and observed SNPs, so the simulated disease loci were included in our data for analysis. Gene × gene interaction was simulated between the DRB1 locus on chromosome 6 and locus A on chromosome 16; the minor allele at locus A (frequency = 0.30) acted in a dominant fashion to increase risk with the DRB1*4 allele (DRB1*4 allele frequency = 0.25). An interaction between locus C on chromosome 6 and sex was simulated with the C allele (frequency = 0.50), acting log-additively to increase risk of RA in females. Women with one copy of the C allele were modeled to have 2.1-fold increased risk versus women with the cc genotype; women with two copies of the C allele showed 4.41-fold increased risk. Locus B on chromosome 8 was modeled to interact with lifelong smoking status in a dominant fashion; smokers with at least one copy of the B allele (B allele frequency = 0.35) had 1.5-fold higher risk of RA versus smokers with the bb genotype. To approximate a case-control study we used control data paired with a single randomly selected affected offspring from each family. One hundred replicates were created by the workshop organizers, no data sets had missing values, and no genotyping error or misclassification of sex and smoking status was assumed. All genotypes were recoded into a pair of binary variables: one as two-allele dominant and the other as two-allele recessive. Smoking status and gender were retained as binary predictors.

The variables of interest (all variables simulated to participate in interactions) included sex, smoking status, and the disease loci on chromosomes 8 and 16. Because of the strong LD between SNPs 153 and 154 on chromosome 6 (D' = 1.0; *r*^2 ^= 0.50) and because the generating model for simulation for the DRB1 disease locus was tri-allelic, we retained both SNPs as variables of interest. To assess the variability of ranking and magnitude of importance measures, we randomly selected a single replicate (number 19) and repeated our analyses using the three ensemble methods 500 times, tallying the ranking and influence measures of each of our 10 variables of interest. GBM tallies rankings only for variables with a non-zero importance score, so any variable with an importance score of 0 does not have a corresponding rank.

## Results

For variables simulated to have a strong effect size (the two SNPs on chromosome 6, dominant and recessive ranking for the first SNP and dominant only for the second SNP), all three methods place these SNPs near the top of the importance rankings (Table 1). In fact, both GBM and MCLR consistently rank these variables in the same position across the interquartile range of 500 runs of the algorithm. RF shows only one ranking position variation in rankings for the two dominant codings of the SNPs on chromosome 6. Only one method ranked recessive coding for the SNP B on chromosome 6 as showing strong association: it had an importance score of 0 across the interquartile range of scores using GBM; MCLR ranked it in the top 10% of variables; and RF ranked this variable sixth consistently within the interquartile range. The dominant coding of the disease locus on chromosome 8 was ranked within the top 15 most important variables by all three methods; even though the effect size of the chromosome 8 × smoking interaction was much smaller than that of the chromosome 6 interactions, the ranking of this variable was stable, varying only one rank between GBM and MCLR and not varying across the interquartile range for RF. The recessive coding for the chromosome 16 disease locus was ranked fairly high in importance by both GBM and RF, with little variation in rankings. Interestingly, MCLR seemed unable to detect this association using importance measures, ranking both codings below the top 20% of all variables in importance. GBM and RF both ranked sex and smoking within the top 10 most important variables, and the rankings did not vary across the interquartile range of scores. MCLR median ranking for sex was two, but the interquartile range was slightly variable from two to six. More interestingly, smoking had a median score of 20, but the interquartile range was rather variable, from 10 to 43.

**Table 1:** Median and interquartile range of variable rankings and importance measures on simulated interacting risk factors

	GBM				MCLR				RF			
			
	Median ranking	Ranking interquartile range	Median importance	Importance interquartile range	Median ranking	Ranking interquartile range	Median importance^a^	Importance interquartile range^a^	Median ranking	Ranking interquartile range	Median importance	Importance interquartile range
Chromosome 6, SNP A, Dominant	1	No variation	43.18	43.08–43.27	4	3–4	1	No variation	2	2–3	110.5	106.8–113.4
Chromosome 6, SNP A, Recessive	3	No variation	9.23	9.20–9.25	3	2–3	1	No variation	4	No variation	55	53.0–57.1
Chromosome 6, SNP B, Dominant	2	No variation	39.02	38.94–39.14	5	4–5	1	No variation	3	2–3	108	105.1–110.4
Chromosome 6, SNP B, Recessive	**--**^b^	**--**	--	--	256	217–300	0.0009628	0.0008214–0.001118	6	No variation	23.4	22.5–24.4
Chromosome 8, Disease Locus, Dominant	13	12–13	0.07	0.065–0.075	7	6–7	0.9998	0.9921–1	14	No variation	2.6	2.5–2.6
Chromosome 8, Disease Locus, Recessive	**--**	**--**	**--**	**--**	21	11–44	0.0105	0.003999–0.428	1322	1079–1533	0.57	0.53–0.61
Chromosome 16, Disease Locus, Dominant	**--**	**--**	**--**	**--**	624	525–716	0.0004029	0.0003219–0.0004992	51	40–75	0.85	0.81–0.91
Chromosome 16, Disease Locus, Recessive	11	No variation	0.087	0.083–0.093	2027	1757–2305	0.0000826	5.63e-5-0.0001214	31	29–32	1.05	1.01–0.12
Sex	5	No variation	1.975	1.96–1.99	2	2–6	0.9998	0.9921–1	8	No variation	11.8	11.6–12.4
Smoking	4	No variation	2.35	2.34–2.36	20	10–43	0.0105	0.003999–0.428	10	No variation	6.8	6.6–7.1

^a^Proportion of times variable selected out of 10,000,000 moves in the Markov chain.

^b^Variable had importance = 0 for median and first and third quartile, so it was not ranked.

Importance scores (as measured by the interquartile range of importance scores for each method) were nearly identical for all variables with non-zero importance scores using GBM, with the largest difference between the first quartile and third quartile score being 0.2 (Table 1). MCLR showed more variability, as expected, because the algorithm simply tallies variables entered into the model at each state of the Markov chain. However, for variables ranked in the top 10 most important variables (chromosome 6 SNPs A (both codings) and the dominant coding of SNP B and sex), the importance measures were stable, either showing the variable was in the model for the entire length of the Markov chain or in 99% to 100% of the models. The variables that had lower rankings showed more variability in importance scores, e.g., the interquartile range for the percent of times smoking was included in the model ranged from 0.004% to 0.428%. RFs were less variable in importance scores than MCLR across both strongly associated and modestly associated variables. The largest difference in the first and third quartiles of importance scores for RF was for the dominant coding of SNP A on chromosome 6 (6.6), and interquartile ranges seemed to scale with the size of the importance scores.

## Conclusion

Overall, the variability of importance rankings and scores using variables involved in modeled interactions was fairly consistent for all methods evaluated for the chromosome 6-sex interaction, which had a much larger effect size than the other two interactions (chromosome 8 × smoking and chromosome 6 × chromosome 16). With regards to the smaller effect sizes, GBM and RF seemed less variable in rankings and importance scores than MCLR, and GBM was superior to RF in stability of rankings and importance measures, which is not surprising because boosting is an averaging process across an additive expansion of trees [13].

It should be noted that these results are specific to this simulation model, and might be very different under alternative simulation models. In particular, the very large effect size for the chromosome 6 locus might paint an overly optimistic picture of the stability of variable importance rankings and scores. This implies that the disease locus on chromosome 8 (smokers carrying at least one copy of the B allele had a 1.5-fold increased risk for RA) in particular may serve as a good benchmark for the variability of statistical learning rankings and importance scores as applied to complex multifactorial diseases when larger effect sizes are also present. In this particular case, it appears that GBM and RF show more stable estimates of variable rankings and importance measures. However, the comparison between RF/GBM and MCLR may not be completely fair, considering that the Gini index used for RF measures the reduction in impurity and GBM attempts to reduce the loss function across the additive expansion of trees, and MCLR is simply tallying variables included in the model across the Markov chain.

Especially in situations in which the expected effect size is modest, researchers who use statistical learning methods to detect SNP × SNP (or gene × gene) interaction in complex disease studies should perform repeated analyses to determine the variability of importance measures and rankings, even for statistical learning algorithms that are thought to be stable.

## Competing interests

The author(s) declare that they have no competing interests.

## References

[B1] Wei Z, Li H (2007). Nonparametric pathway-based regression models for analysis of genomic data. Biostatistics.

[B2] R Development Core Team R: A language and environment for statistical computing. http://www.R-project.org.

[B3] Kooperberg C, Ruczinski I, LeBlanc ML, Hsu L (2001). Sequence analysis using logic regression. Genet Epidemiol.

[B4] Ruczinski I, Kooperberg C, LeBlanc ML (2003). Logic regression. J Comput Graph Stat.

[B5] Kooperberg C, Ruczinski I (2005). Identifying interacting SNPs using Monte Carlo logic regression. Genet Epidemiol.

[B6] Kooperberg C, Ruczinski I The LogicReg Package. R package version 141.

[B7] Breiman L, Friedman JH, Olshen RA, Stone CJ (2004). Classification and Regression Trees.

[B8] Breiman L (2001). Random forests. Mach Learn.

[B9] Liaw A, Weiner M The randomForest Package. R package version 45-16.

[B10] Freund Y, Schapire RE (1997). A decision-theoretic generalization of online learning and an application to boosting. J Comput System Sci.

[B11] Friedman JH (2001). Greedy function approximation: a gradient boosting machine. Ann Stat.

[B12] Ridgeway G The gbm package. R package version 15-7.

[B13] Friedman JH, Lyons L, Mount R, Reitmyer R (2003). Recent advances in predictive (machine) learning. Statistical Problems in Particle Physics, Astrophysics and Cosmology, Proceedings of the PHYSTAT 2003 Conference: 8–11 September 2003; Palo Alto.

